# Stress is associated with larger perivascular spaces in depression: A 7-tesla MRI study

**DOI:** 10.1192/j.eurpsy.2021.890

**Published:** 2021-08-13

**Authors:** D. Ranti, P. Balchandani, L. Morris

**Affiliations:** Psychiatry, Icahn School of Medicine at Mount Sinai, New York, United States of America

**Keywords:** perivascular spaces, trauma, Depression, high-field MRI

## Abstract

**Introduction:**

Emerging evidence in depressive phenotypes suggests that the breakdown of the blood brain barrier (BBB) andhigh levels of inflammatory cytokines in states of persistent stress or traumatic experiences may contribute to its pathophysiology. Ultra-high field MRI may aid in the radiological detection of maladaptations of the glymphatic system related to BBB integrity that may not be visualized at lower field strengths.

**Objectives:**

We aimed to investigate the link between glymphatic neuroanatomy in the form of perivascular spaces (PVS) and trauma experience in patients with major depressive disorder.

**Methods:**

We examined PVS’s in patients with major depressive disorder and in healthy controls using 7-Tesla MRI and a semi-automated segmentation algorithm.

**Results:**

After controlling for age and gender, we found that the number of traumatic life events experienced was positively correlated with total PVS volume in MDD patients (r= 0.50, p= 0.028) and the overall population (r= 0.34, p= 0.024). Furthermore, the number of traumatic events eliciting fear, helplessness, or horror was positively correlated with total PVS volume in MDD patients (r= 0.50, p= 0.030) and the overall population (r= 0.32, p= 0.023). As expected, age correlated positively with PVS count (r= 0.37, p= 0.013), PVS total volume (r= 0.53, p< 0.001), and PVS density (r= 0.68, p< 0.001 in all participants.

**Conclusions:**

These results suggest a relationship between glymphatic dysfunction potentially related to BBB integrity and psychological trauma in patients with depression, and suggest that glymphatic impairment may play a role in trauma-related symptomatology.

